# The Upper Limits of Risk Ratios and Recommendations for Reporting Risk Ratios, Odds Ratios, and Rate Ratios

**DOI:** 10.7759/cureus.37799

**Published:** 2023-04-18

**Authors:** Yi-Sheng Chao, Chao-Jung Wu, June Y Po, Shih-Yu Huang, Hsing-Chien Wu, Hui-Ting Hsu, Yen-Po Cheng, Yi-Chun Lai, Wei-Chih Chen

**Affiliations:** 1 Epidemiology and Public Health, Independent Researcher, Montreal, CAN; 2 Computer Science, Université du Québec à Montréal, Montreal, CAN; 3 Natural Resources Institute, University of Greenwich, London, GBR; 4 Anesthesiology, Shuang Ho Hospital, New Taipei, TWN; 5 Internal Medicine, National Taiwan University Hospital Jinshan Branch, New Taipei, TWN; 6 Pathology, Changhua Christian Hospital, Changhua, TWN; 7 Neurological Surgery, Changhua Christian Hospital, Changhua, TWN; 8 Chest Medicine, National Yang Ming Chiao Tung University Hospital, Yilan, TWN; 9 Chest Medicine, Taipei Veterans General Hospital, Taipei, TWN

**Keywords:** 95% confidence interval, odds ratios, upper limits, rate ratios, relative measures, risk ratios

## Abstract

Background

Relative measures, including risk ratios (RRs) and odds ratios (ORs), are reported in many epidemiological studies. RRs represent how many times a condition is likely to develop when exposed to a risk factor. The upper limit of RRs is the multiplicative inverse of the baseline incidence. Ignoring the upper limits of RRs can lead to reporting exaggerated relative effect sizes.

Objectives

This study aims to demonstrate the importance of such upper limits for effect size reporting via equations, examples, and simulations and provide recommendations for the reporting of relative measures.

Methods

Equations to calculate RRs and their 95% confidence intervals (CIs) were listed. We performed simulations with 10,000 simulated subjects and three population variables: proportions at risk (0.05, 0.1, 0.3, 0.5, and 0.8), baseline incidence (0.05, 0.1, 0.3, 0.5, and 0.8), and RRs (0.5, 1.0, 5.0, 10.0, and 25.0). Subjects were randomly assigned with a risk based on the set of proportions-at-risk values. A disease occurred based on the baseline incidence among those not at risk. The incidence of those at risk was the product of the baseline incidence and the RRs. The 95% CIs of RRs were calculated according to Altman.

Results

The calculation of RR 95% CIs is not connected to the RR upper limits in equations. The RRs in the simulated populations at risk could reach the upper limits of RRs: multiplicative inverse of the baseline incidence. The upper limits to the derived RRs were around 1.25, 2, 3.3, 10, and 20, when the assumed baseline incidence rates were 0.8, 0.5, 0.3, 0.2, and 0.05, respectively. We demonstrated five scenarios in which the RR 95% CIs might exceed the upper limits.

Conclusions

Statistical significance does not imply the RR 95% CIs not exceeding the upper limits of RRs. When reporting RRs or ORs, the RR upper limits should be assessed. The rate ratio is also subject to a similar upper limit. In the literature, ORs tend to overestimate effect sizes. It is recommended to correct ORs that aim to approximate RRs assuming outcomes are rare. A reporting guide for relative measures, RRs, ORs, and rate ratios, is provided. Researchers are recommended to report whether the 95% CIs of relative measures, RRs, ORs, and rate ratios, overlap with the range of upper limits and discuss whether the relative measure estimates may exceed the upper limits.

## Introduction

There are several statistical measures of association that aim to quantify the strengths of the associations. Relative measures of associations include risk ratios (RRs), odds ratios (ORs), and rate ratios [1,2]. The RR is one of the relative measures widely used to quantify disease risks [1,2]. In particular, it is used to quantify the impacts of health hazards on the occurrence of diseases, compared with those not exposed to the hazards [1,3,4]. Among relative measures, RRs are simple to interpret and often used in randomized controlled trials and cohort studies [1,4,5]. The RR is calculated by dividing the risk in the at-risk group by the risk in the control group and represents how many times it is likely to develop a condition when exposed to a risk factor [2]. The OR, another relative measure, is generally used in case-control studies to approximate RRs [2]. When the prevalence of the outcome is low, ORs approach RRs [6].

However, there are limitations to the RR. First, the ranges of RRs have not been well discussed in major epidemiology textbooks [2,7,8]. In a few textbooks, the RR is considered to range from 0 to infinity [7]. It is correct that the RR collected from a large number of empirical studies can have a range of less than 1 to 25 or more [9]. Nevertheless, for a single given study, upper limits to RRs are the multiplicative inverse of the baseline incidence [10] and mathematical limits to the RRs in a population with well-defined baseline cumulative incidence [7] (also called incidence in a cohort study setting [1], incidence proportion [2], or risk [11]). As the baseline incidence is close to 1, the upper limits of RRs approach 1. Meaning, as the general population’s disease baseline incidence approaches 1, everyone carries the disease and the risk of disease in a particular “comparison” group will be similar to the “control” group drawn from the baseline population. With the same risk in the comparison and control group, the upper limits of the RR would approach 1. The upper limit of RRs may be confused with the upper bound of a 95% confidence interval (CI) of the RR. However, the 95% CIs are ranges for statistical inferences [6]. The relationship between the 95% CIs and upper limits of RRs has not been discussed in major epidemiology textbooks [2,7,8] or in major review articles [10,12].

The second limitation is that the RR is a relative measure of effects. It has been recommended that reports of relative effects should also include measures of absolute effects, for example, reporting the absolute risk differences and the RRs between two groups when measuring the effect sizes of risk factors [4,13]. Both relative and absolute effects are needed for research users to readily ascertain both the statistical and clinical significance of the risk differences.

Another limitation is that the RR alone is not enough to describe the impact of the related risk factor in a given population. An additional parameter, “proportions at risk,” is important that can modify the relationships between RRs and disease incidence [5]. In a population, it is likely that only some of the people are exposed to health hazards and their incidence of developing disease increases proportionally to the RRs of the hazards. The proportions of the population at risk can influence the absolute number of individuals who develop the outcome [5]. The interplay of the factors introduced above has not been well appreciated and perceived in major reporting guidelines [14]. It is also challenging for primary studies to assess their interplay. For example, a cohort study that examines an intervention by enrolling a comparison and a control group would estimate one RR per intervention. It is difficult to explore the upper limits of RRs and the relationship between RRs, baseline incidence, and proportions at risk using empirical studies. A simulation model can provide a simple and easy-to-understand illustration of how these factors can influence overall incidence rates [5]. The other limitations can be found in other textbooks [2,7,8] or review articles [10,12].

In this study, we aim to demonstrate the interplay between disease occurrence, risk factor, and proportions at risk in equations by establishing a pilot simulation model, as well as examine the impact of the upper limits of RRs, which can be relevant in future research. In addition, we provide recommendations on the reporting of relative measures (RRs, ORs, and rare ratios) based on the results.

## Materials and methods

Simulation model development

**Figure 1:**
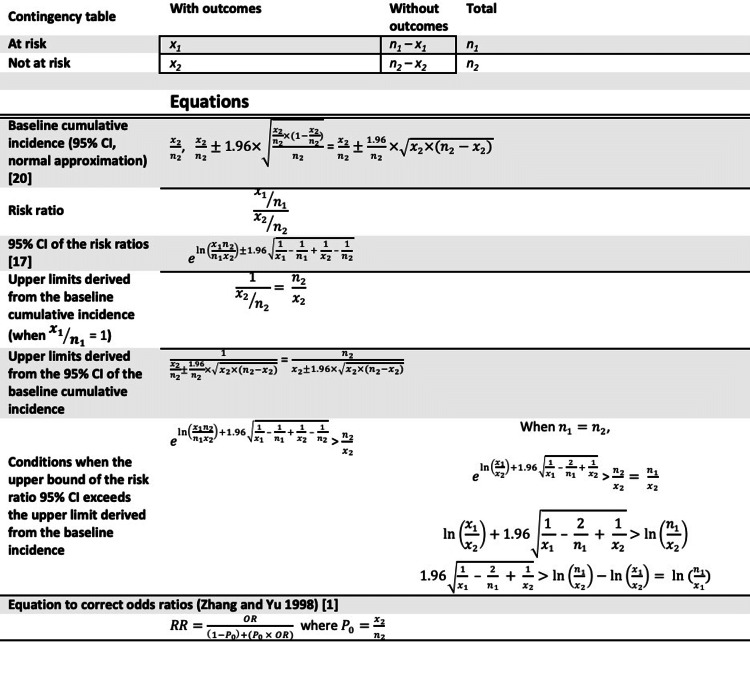
Equations for risk ratio calculation, 95% confidence intervals (CIs), risk ratio upper limits, and odds ratio correction.

In a basic model, we assumed that there is only one risk factor for the disease and that the disease occurs at random in a cohort (Table 1 and Figure 1). In Eq. 1, the overall cumulative incidence (denoted by I) in a population is estimated via a function of three factors in the model: the proportions of the population at risk (denoted by Pr, ranging from 0 to 1), the baseline cumulative incidence among individuals not at risk in the population (denoted by IR, ranging from 0 to 1), and the RRs resulting from the risk factor (denoted by RR).

**Table 1:** Risk ratio, proportions at risk, and disease incidence. Equations consider a single disease risk factor and assume the disease occurs at random in the populations at risk and not at risk, based on the at-risk incidence and baseline incidence, respectively. Pr = proportions at risk; RR = risk ratio; IR = baseline cumulative incidence.

	Proportion at risk	Proportion not at risk
Proportions relative to all subjects	Pr	1 - Pr
Risk ratio	RR	1 by definition
Cumulative incidence	RR x IR	IR
Overall incidence	Pr x RR x IR + (1 - Pr) x 1 x IR = IR + Pr x RR x IR - Pr x IR	
Scenario 1-1		
Incidence reaching 1 in the population not at risk	RR x IR = 1 (max)	IR = 1
Derived risk ratio	(RR x IR)/IR = 1 / 1	1 by definition
Overall incidence	Pr x RR x IR + (1 - Pr) x 1 x IR = Pr x 1 x 1 + (1 – Pr) x 1 = 1	
Scenario 1-2		
Incidence reaching 1 in the population at risk	RR x IR = 1 (max)	IR < 1
Derived risk ratio	1/IR	1 by definition
Overall incidence	Pr x RR x IR + (1 - Pr) x 1 x IR = Pr x 1 + (1 – Pr) x 1 x IR = Pr + IR – Pr x IR	

Estimation of the overall disease incidence: I = Pr x RR x IR + (1 - Pr) x 1 x IR = IR + Pr x RR x IR - Pr x IR (Eq. 1).

**Figure 2:**
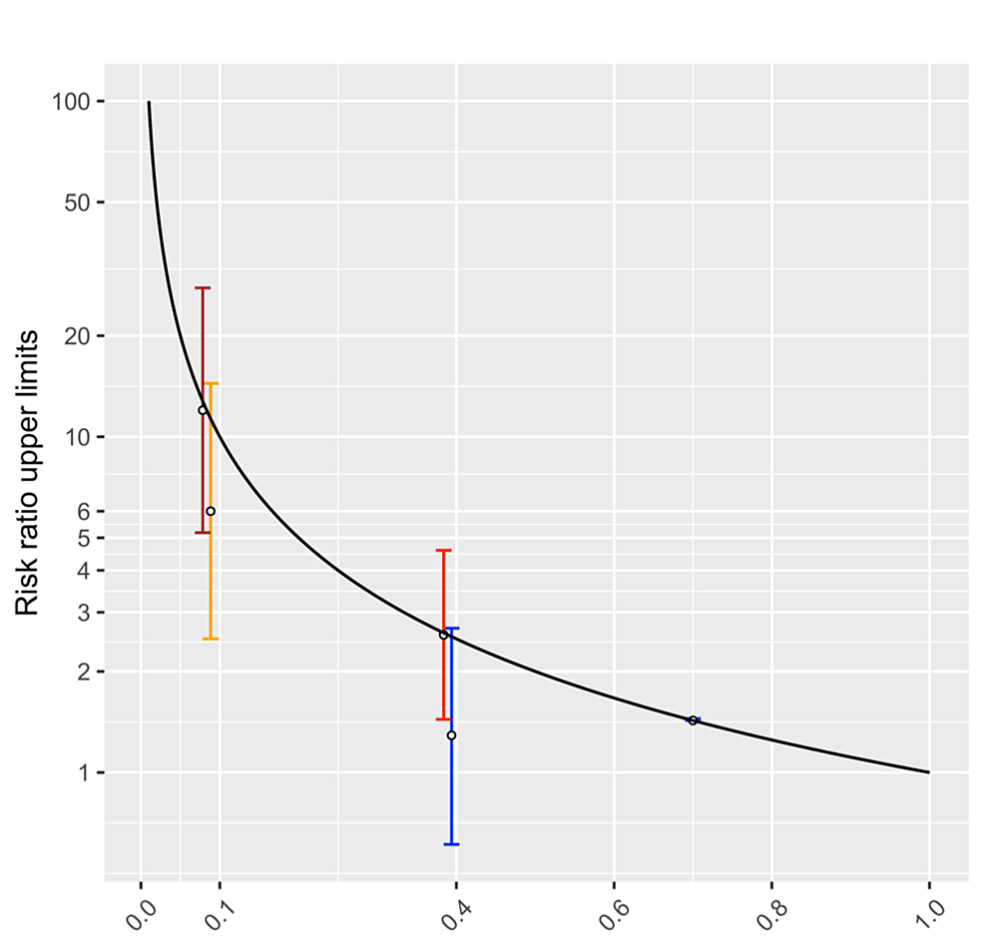
The upper limits of risk ratios based on the baseline cumulative incidence (point estimates) among those not at risk. Black line = the upper limits to risk ratios according to the baseline cumulative incidence; blue line = Scenario 3-1; red line = Scenario 3-2; orange line = Scenario 3-3; brown line = Scenario 3-4; circle with 0.7 cumulative incidence = Scenario 3-5.

In Scenario 1-1 in Table 1, the baseline cumulative incidence (IR) reaches 1, and, no matter how effective the risk factor may be, the RR is 1 among the individuals at risk and the overall incidence is 1. In Scenario 1-2, the incidence in the population at risk reaches 1 (IR x RR = 1) and the baseline incidence remains less than 1. No matter how large the effect of the risk factor may be, the RR will be 1/IR at most. For example, when the baseline cumulative incidence (IR) is 0.1, 0.2, 0.5, or 0.8, the upper limits of the RRs will be 10, 5, 2, or 1.25, respectively. The upper limits of the RRs that can be reached in the populations at risk are plotted against the baseline cumulative incidence in the control group (not at risk) in Figure 2. This demonstrates the undeclared upper limits to the values of RRs. This relationship will become more complex when we consider more than one disease or risk factor in the estimation.

Simulation model assumption and specification

A published simulation model was adopted for this study [5]. The assumptions and the parameters of the micro simulations are listed in Table 2. In detail, simulated populations were created based on pre-specified parameters for each combination of assumed proportions at risk (Pr), baseline cumulative incidence (IR), and RRs. Each combination of parameters was used to create a simulated population. For each simulation, one risk factor directly influences the incidence of the disease. Disease statuses and risk exposure were classified accurately. Those not at risk were randomly assigned to the disease based on the assumed baseline incidence (IR). The incidence among the subjects at risk was the product of the baseline incidence and the assumed RRs (IR x RR). There were 10,000 simulated individuals created for each simulation. Due to random assignment, the observed incidence was variable. The assumed values and the observed incidence rates of both at-risk and not-at-risk groups were recorded and the RRs in simulated populations were calculated. The proportions at risk (Pr) were assumed to be 0.05, 0.1, 0.3, 0.5, and 0.8. The baseline cumulative incidence (IR) of developing the disease for those not affected by the risk factor were 0.05, 0.1, 0.3, 0.5, and 0.8. The RRs of developing the disease if at risk were 0.5 (less likely to develop the disease), 1.0 (equally likely to develop the disease), 5.0, 10.0, and 25.0 (multiple times more likely to develop the disease). Since the maximum incidence rate is 100%, the products of the incidence rates and risk ratios (IR x RR) could not exceed 1. There were 125 (5 x 5 x 5) combinations of the proportions at risk (Pr), baseline incidence (IR), and RRs. We conducted 10 simulations for each combination and calculated the summary statistics, such as mean values and variances. For example, 10 simulations were conducted with Pr as 0.1, IR as 0.3, and RR as 5.0, one of the combinations of the three parameters. All statistical analyses were conducted under R environment (v3.5.1, Vienna, Austria) [15] and RStudio (v1.1.463, RStudio, Inc., Boston, MA) [16]. All the R codes are in Appendix 1 and are available from the corresponding author upon reasonable request.

**Table 2:** The assumptions and the assessments of the simulated disease.

	Assumptions	
1	One risk factor of interest directly influenced the disease occurrence	
2	Disease statuses and risk exposure classified accurately	
3	Disease status randomly assigned to the individuals based on the assumed incidence rates and risk ratios	
	Parameters of disease occurrence simulations	
1	Population sizes	10,000
2	Proportions at risk	0.05, 0.1, 0.3, 0.5, and 0.8
3	Baseline cumulative incidence rates of the disease	0.05, 0.1, 0.3, 0.5, and 0.8
4	Risk ratios of developing disease	0.5, 1, 5, 10, and 25
5	Number of simulations for each combination of proportions at risk, baseline cumulative incidence, and risk ratios	10
6	The maximal of the products of cumulative incidence and risk ratios equal to 1	
	Statistics for assessment	
1	Derived risk ratios	
2	Derived cumulative incidence	

95% CIs of risk ratios

Mathematically, RRs ranged from 0 (baseline incidence with any non-zero value and no disease occurrence among those at risk) to infinity (baseline incidence as 0 and all at-risk individuals diseased). The upper limits of RRs are not the same as the upper bounds of 95% CIs. Unlike the upper limits of RRs, which are mathematical limits in a population with well-defined baseline incidence, the 95% CIs are ranges for point estimates to estimate uncertainties in statistical inference [1,17]. It is important to determine whether their 95% CIs include the null hypothesis, usually whether RRs equal 1. The 95% CIs of the RRs were estimated according to Altman (1991) in Figure 1 [6]. The exponentiation of the log values of RRs added with 1.96 times the square root of the sum of the multiplicative inverse of the numbers of individuals in four cells of the contingency tables is the upper bound of the 95% CI [6]. This equation is listed in Figure 1 [6]. The conditions when the upper bound of the RR 95% CI exceeds the upper limit derived from the baseline incidence were also written in equations. The 95% CIs of RRs are derived from the exponentiation of Euler's number (e) and thus remain positive numbers [6]. The p-values and 95% CIs of RRs were confirmed with public websites [18,19].

Range of upper limits derived from baseline incidence 95% CIs

The baseline cumulative incidence in a cohort study has a 95% CI to describe its uncertainty [20]. The 95% CIs could be obtained assuming normal approximation [20]. An upper limit can be derived from the multiplicative inverse of the baseline cumulative incidence (point estimate) and a range of upper limits can be derived from the 95% CI of the incidence. The ranges of RR upper limits derived from the 95% CIs of the baseline cumulative incidence in the five scenarios in Table 3 were assessed for their relationships with the RR 95% CIs.

**Table 3:** Risk ratio calculation, 95% confidence intervals, and scenarios with risk ratio 95% CIs exceeding risk ratio upper limits. CI = confidence interval; H0 = null hypothesis; OR = odds ratio; RR = risk ratio. Scenario 3-1 = blue line in Figure 2; Scenario 3-2 = red line in Figure 2; Scenario 3-3 = orange line in Figure 2; Scenario 3-4 = brown line in Figure 2; Scenario 3-5 = black vertical line in Figure 2. Risk ratio confidence intervals calculated by the authors according to Altman (1991) [6] and confirmed with the results from public websites (https://www.medcalc.org/calc/relative_risk.php and https://www.scalelive.com/relative-risk.html).

Scenario 3-1			
	With outcomes	Without outcomes	Total
At risk	9	9	18
Not at risk	7	11	18
Baseline cumulative incidence (95% CI)	7/18 = 0.39 (0.16 to 0.61)		
RR (95% CI)	1.29 (0.61 to 2.69)		
Significance of RR (P, H_0_: RR = 1)	0.50		
Upper limits of the risk ratios (when at-risk incidence = 1)	(18/18)/(7/18) = 1/(7/18) = 18/7 = 2.57		
Range of upper limits derived from incidence 95% CIs	1/0.16 to 1/0.61 = 1.63 to 6.11		
Scenario 3-2			
	With outcomes	Without outcomes	Total
At risk	18	0	18
Not at risk	7	11	18
Baseline cumulative incidence (95% CI)	7/18 = 0.39 (0.16 to 0.61)		
RR (95% CI)	2.57 (1.44 to 4.59)		
Significance of RR (P, H_0_: RR = 1)	0.0014		
Upper limits of the risk ratios (when at-risk incidence = 1)	(18/18)/(7/18) = 1/(7/18) = 18/7 = 2.57		
Range of upper limits derived from incidence 95% CIs	1/0.16 to 1/0.61 = 1.63 to 6.11		
Scenario 3-3			
	With outcomes	Without outcomes	Total
At risk	30	30	60
Not at risk	5	55	60
Baseline cumulative incidence (95% CI)	5/60 = 0.08 (0.01 to 0.15)		
RR (95% CI)	6.00 (2.50 to 14.42)		
Significance of RR (P, H_0_: RR = 1)	<0.001		
Upper limits of the risk ratios (when at-risk incidence = 1)	(60/60)/(5/60) = 1/(5/60) = 60/5 = 12.00		
Range of upper limits derived from incidence 95% CIs	1/0.15 to 1/0.013 = 6.5 to 74.6		
Scenario 3-4			
	With outcomes	Without outcomes	Total
At risk	60	0	60
Not at risk	5	55	60
Baseline cumulative incidence (95% CI)	5/60 = 0.08 (0.01 to 0.15)		
RR (95% CI)	12.00 (5.18 to 27.77)		
Significance of RR (P, H_0_: RR = 1)	<0.001		
Upper limits of the risk ratios (when at-risk incidence = 1)	(60/60)/(5/60) = 1/(5/60) = 60/5 = 12.00		
Range of upper limits derived from incidence 95% CIs	1/0.15 to 1/0.013 = 6.5 to 74.6		
Scenario 3-5			
	With outcomes	Without outcomes	Total
At risk	10,000	0	10,000
Not at risk	7,000	3,000	10,000
Baseline cumulative incidence (95% CI)	7,000/10,000 = 0.7 (0.69 to 0.71)		
RR (95% CI)	1.43 (1.41 to 1.447)		
Significance of RR (P, H_0_: RR = 1)	<0.001		
Upper limits of the risk ratios (when at-risk incidence = 1)	(10,000/10,000)/(7,000/10,000) = 1/(7,000/10,000) = 1.43		
Range of upper limits derived from incidence 95% CIs	1/0.71 to 1/0.69 = 1.41 to 1.447		

Scenarios with an overlap between the risk ratio 95% CIs and the range of upper limits

Five scenarios (3-1 to 3-5) were created to show the overlap between the RR 95% CIs and the upper limits (Table 3). Because the RRs and the numbers of individuals in the four groups in the contingency tables can simultaneously influence the RR 95% CIs, it was not possible to show all possible ranges of RR 95% CIs in Figure 2. For simplicity and practicality, the numbers of observations were assumed to be equal in two risk groups in each of the first four scenarios. Scenario 3-1 was designed to have a statistically insignificant RR and Scenarios 3-2 to 3-5 to have statistically significant RRs to demonstrate the lack of strong connection between RR statistical significance and the overlap between RR 95% CIs and the range of upper limits. Scenarios 3-2, 3-4, and 3-5 demonstrated the situations in which the point estimates of RRs were reaching the upper limits derived from the baseline incidence. In the three scenarios, the upper bounds of RR 95% CIs also exceeded the upper limits derived from the baseline disease incidence. Scenario 3-5 demonstrated that the RR 95% CI completely overlapped with the range of upper limits derived from the 95% CI of baseline incidence.

## Results

Simulation accuracy based on assumed parameters

**Figure 3:**
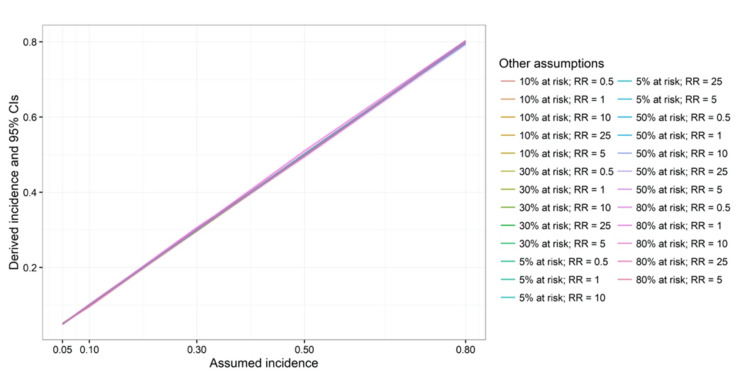
Assumed and derived cumulative incidence among individuals not at risk. CI = confidence interval; RR = risk ratio.

**Figure 4:**
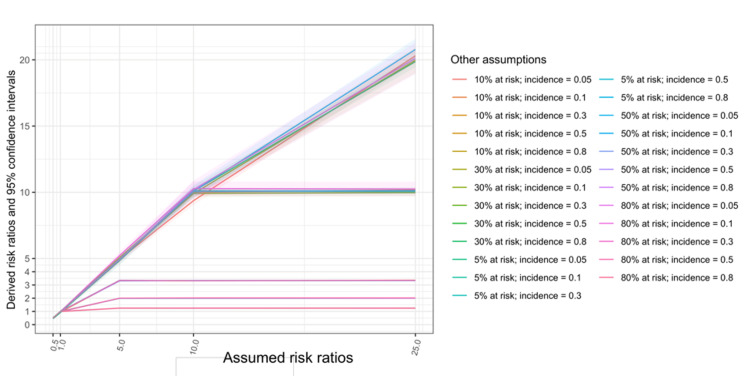
Assumed and derived risk ratios of disease development based on different assumed proportions at risk and baseline incidence. The risk ratios derived from simulated populations are similar to the values assumed when the upper limits of the risk ratios are not met. The upper limits of risk ratios are the multiplicative inverse of the cumulative incidence in the control (not at risk) groups when the cumulative incidence in the at-risk group reaches 1.

The assumed baseline incidence rates are similar to the derived baseline incidence rates of individuals not at risk (Figure 3). This suggests that the simulations were accurately implemented based on the assumed proportions at risk and baseline incidence (see Appendix 2). In Figure 4, the assumed RRs are plotted against the derived ones. When the assumed baseline incidence rates were 0.8, 0.5, 0.3, 0.2, and 0.05, the upper limits of the RRs were around 1.25, 2, 3.3, 10, and 20, respectively (upper limits also shown in Figure 2). In addition, the simulation model shows that varying the assumed proportions at risk did not affect the derived RRs.

Ranges of upper limits derived from the 95% CIs of baseline cumulative incidence

**Figure 5:**
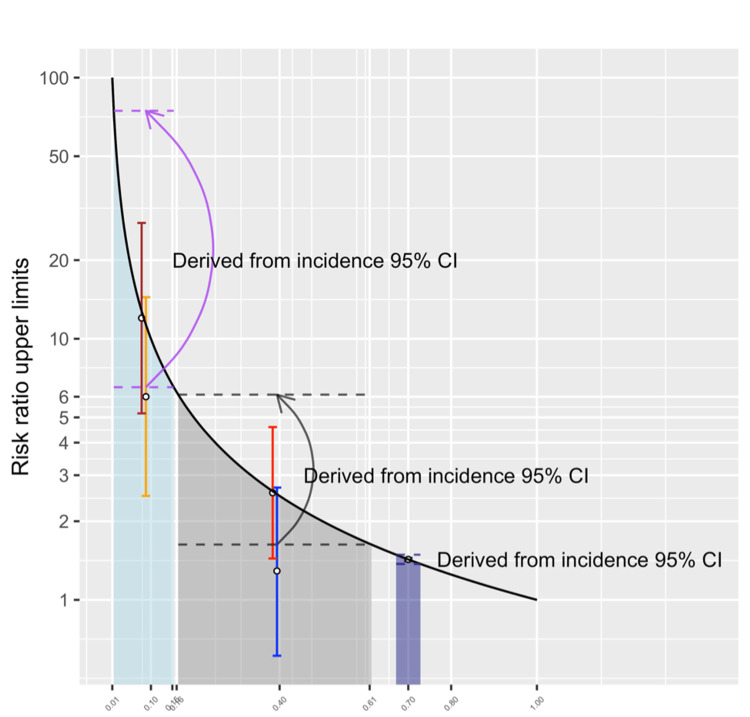
The upper limits of risk ratios derived from the baseline incidence 95% CIs in four cases. CI = confidence interval; RR = risk ratio. Blue or gray shade = 95% CIs of the baseline cumulative incidence. Black solid line = upper limits to risk ratios according to the baseline cumulative incidence; dashed lines = upper limits of RRs derived from the upper or lower bounds of the baseline cumulative incidence 95% CIs. Gray curve = the range of risk ratio upper limits derived from the 95% CIs of the baseline cumulative incidence in Scenarios 3-1 and 3-2. Purple curve = the range of risk ratio upper limits derived from the 95% CIs of the baseline cumulative incidence in Scenarios 3-3 and 3-4. Blue line = Scenario 3-1; red line = Scenario 3-2; orange line = Scenario 3-3; brown line = Scenario 3-4; black vertical line = Scenario 3-5. All scenarios are shown in Table 3.

There were uncertainties to the baseline cumulative incidence in Scenarios 3-1 to 3-5. The 95% CIs of the baseline incidence were calculated in Table 3 [20]. The ranges of upper limits derived from the baseline incidence 95% CIs were also listed in Table 3 and plotted in Figure 5. The curves in Figure 5 indicated the upper limits derived from the baseline incidence 95% CIs with the arrows pointing to the upper limits derived from the lower bounds of the baseline incidence 95% CIs. There was an overlap between the range of upper limits and the RR 95% CIs in Scenarios 3-1 to 3-5.

Risk ratio 95% CIs and ranges of risk ratio upper limits

In the simulated populations, our findings show a disconnect between the upper limits of RRs and their CIs [6]. There might be an overlap between the RR 95% CIs and the upper limits derived from the baseline incidence. In other words, the RR upper limits and the RR 95% CI were not significantly different. Due to the difficulties in visually presenting all CIs calculated from possible baseline incidence and RR combinations, we provided five scenarios in which there was an overlap between the RR 95% CIs and the range of RR upper limits derived from baseline incidence 95% CIs (Figure 2 and Table 3). The baseline incidence 95% CIs were calculated by authors and confirmed by two public websites [18,19]. In Scenarios 3-1 to 3-4, there were equal numbers of individuals at risk and not at risk in each scenario, specifically 18 individuals per risk status in both Scenario 3-1 and 3-2, and 60 individuals in Scenario 3-3 and 3-4. In Scenario 3-1, the RR was 1.29 (95% CI = 0.61 to 2.69) and the upper limit to the RR was 2.57 (baseline IR = 7/18 = 0.39 (95% CI = 0.16 to 0.61); upper limit = 1/IR = 18/7 = 2.57 (range derived from baseline incidence 95% CI = 1.63 to 6.11, the same definition below)). In Scenario 3-2, the distribution of those not at risk was the same as in Scenario 3-1, but the RR (point estimate) reached the upper limit derived from the baseline incidence point estimate because all of the individuals at risk presented the disease. The RR was exactly the multiplicative inverse of the baseline incidence, i.e., 2.57 (baseline IR = 7/18 (95% CI = 1.44 to 4.59); upper limit = 2.57 (range = 1.63 to 6.11)). The point estimate of RR matched the upper limit and more than half of the RR 95% CI exceeded the upper limit.

In Scenario 3-3, the RR was 6.0 (95% CI = 2.5 to 14.42) and the upper limit was 12.0 (IR = 5/60; upper limit = 1/IR = 60/5 = 12.0, range = 6.5 to 74.6). In Scenario 3-4, the distribution of those not at risk was the same as in Scenario 3-3, but the RR point estimate also reached the upper limit because all of the individuals at risk presented the outcome. The RR was exactly the multiplicative inverse of the baseline incidence, 12.0 (baseline IR = 5/60; upper limit = 12.0). However, the 95% CI ranged from 5.18 to 27.77 and there was an overlap with the range of upper limits derived from baseline incidence 95% CI (6.5 to 74.6). In Scenario 3-5, the RR was 1.43 (95% CI = 1.41 to 1.447) and the upper limit to the RR was also 1.43 (IR = 7,000/10,000; upper limit = 1/IR = 10,000/7,000 = 1.43, range = 1.41 to 1.447). The RR 95% CI completely matched the range of upper limits derived from baseline incidence 95% CI. The RR in Scenario 3-1 was not statistically significant (p = 0.50), but the RR was significant in Scenarios 3-2 to 3-5 (p < 0.05 for all).

## Discussion

To our knowledge, this is the first study to demonstrate the interaction between RR 95% CIs and the upper limits to RRs. The upper limits to RRs in individual studies have not been well perceived in major epidemiology textbooks [2,20]. We have provided equations and simulation results to show the upper limits to RRs and examples to illustrate RR point estimates, RR 95% CIs, RR upper limits, and the ranges of RR upper limits derived from baseline incidence 95% CIs. The upper limits to RRs could be well illustrated in simulations and match the expectations in equations. Because the at-risk incidence cannot exceed 1, the largest RR can be obtained in a single study is the multiplicative inverse of the baseline incidence in the control group [10]. Based on the equations, we provided five scenarios with contingency tables in which the RR 95% CIs overlap with the ranges of upper limits derived from baseline incidence 95% CIs. This overlap has important implications in clinical research discussed below. These scenarios also show that statistical significance does not guarantee that RR would not exceed the upper limits.

Disconnect between risk ratio 95% CIs and upper limits

The simulations and findings in this study provide several implications for research methods that involve relative measures, particularly RRs and ORs. Greater consideration on the upper limits of RRs is needed when methods involve relative measures, although their implications have not been well perceived in the literature. The upper limits can be understood mathematically [10] or visually via simulations. In the real world, researchers can rarely observe multiple RRs and explore the upper limits of RRs in their trials, because two groups in a trial can only provide one RR estimate. Hence, primary studies focus mostly on the RR 95% CIs to determine statistical significance. We have demonstrated that the calculation of RR 95% CIs [6] is not well connected to the upper limits of RRs. Although 95% CIs are derived from well-established methods [6], ignoring this upper limit can exaggerate the possible extent of the effect sizes and maintain excessive reliance on relative measures. Hence, reports of absolute risk measures, such as risk differences, and the upper limits derived from baseline incidence are recommended alongside relative measures.

Recommendations on relative measure reporting

**Figure 6:**
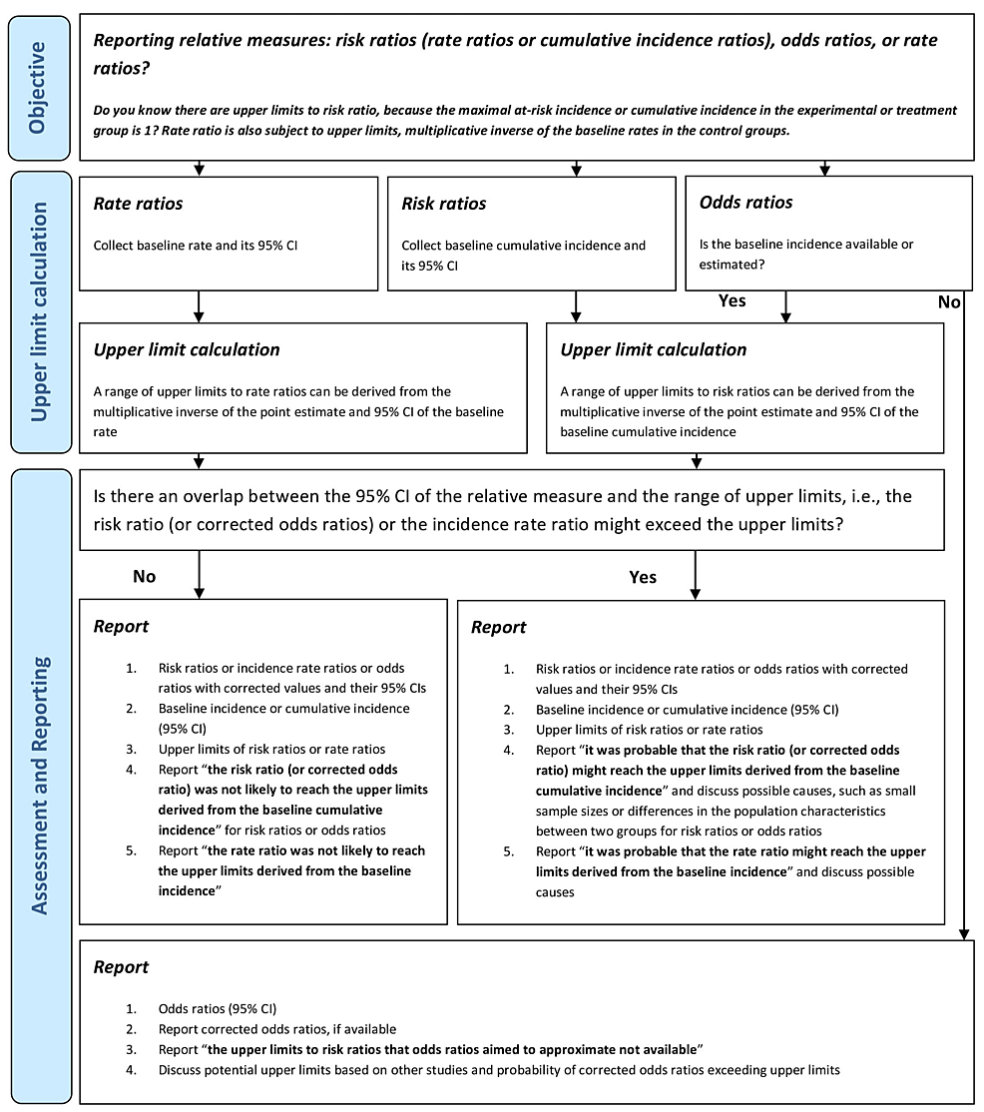
Recommended reporting of relative risk measures.

There are further implications on reporting, power analysis, and the use of other relative measures, due to the disconnect between the 95% CIs and the upper limits of RRs. We argue that specific guidance is needed for researchers to report relative effect sizes for their studies and for research users to understand the limitations of these measures. We provide the following recommendations. First, the reporting of effect sizes should be improved. This is not only concerning the lack of reporting absolute changes [4] but also the inadequate attention to the constraints of relative measures. We recommend that researchers report the absolute and relative effect sizes, and also describe whether there is an overlap between the RR 95% CIs and the range of upper limits derived from the baseline incidence 95% CI. A guide for relative measure reporting is in Figure 6.

Take RRs for example, researchers are expected to collect information on RRs with their 95% CIs, baseline incidence with its 95% CI, and upper limits derived from baseline incidence and its 95% CI. A rule of thumb for the statistical significance in the difference between two 95% CIs is to examine whether there is an overlap in the 95% CIs [21,22]. While there is an overlap between the RR 95% CI and the range of upper limits derived from baseline incidence 95% CI, it is important to understand that the RR may not be significantly different from the upper limits. Researchers are advised to state that there is a probability that the RR 95% CI might reach the upper limits derived from the baseline incidence and to discuss the possible causes, such as small sample sizes or differences in the population characteristics or sampling methods in two groups. Reporting RRs and 95% CIs that may exceed the upper limits can exaggerate the effect sizes. The impact of a potential overestimation of RRs should also be discussed. Without an overlap, the RRs may be significantly different from the upper limits and thus are not likely to exceed the upper limits. Researchers can report that the RRs were not likely to reach the upper limits derived from the baseline cumulative incidence.

Second, the simulations also demonstrated that as the baseline incidence approaches 1 in a cohort, the upper limits of RRs also approach 1. While reporting trial results, RRs close to 1 may make researchers less inclined to examine whether RR 95% CIs reach the upper limits. However, the upper limits approach 1 as the baseline incidence approximates 1. Studies conducted in populations with high baseline incidence should pay more attention to the upper limits of RRs and explore whether the 95% CIs of RRs may be unrealistic, i.e., the RR may reach upper limits because the 95% CI and the range of upper limits overlap.

Third, the statistical significance to examine whether the RRs are different from the null hypothesis should not be used to assess whether the RRs exceed the upper limits. In the scenarios in Table 3, we demonstrated that statistically significant RRs can also have an overlap between the 95% CIs and the range of upper limits derived from baseline incidence 95% CI. We do not recommend researchers to use statistical significance as the guide to judge the relationship between RRs and their upper limits.

Fourth, power analysis for the estimation of the sample sizes in clinical trials and cohort studies can be conducted with expected RRs [23,24]. Some methods require researchers to specify target RRs before trial implementation [23-25]. Without acknowledging the upper limits of RRs, researchers may target RRs exceeding their upper limits. For example, targeting a RR of 2 in a population with a baseline cumulative incidence rate of 0.55 that has a RR upper limit of 1.82 (1/0.55). This can lead to an underestimation of the required sample sizes and subsequently inadequately powered studies. We strongly recommend avoiding the use of power analysis tools that solely depend on the expected RRs. For example, one of the online calculators requires users to enter the proportions of the exposure within the control group and the RRs associated with exposure [26]. When implausible RRs (higher than the upper limits) are assumed, it does not report this upper limit and only warns about unreliable analysis due to low expected counts [26]. We recommend that the upper limits of RRs can be explicitly considered in similar tools and the upper limits can be reported in the warning messages.

We also recommend the assessment of the potential upper limits of RRs before conducting trials. With an estimated baseline incidence of 0.39 in a group of 18 participants, the range of potential upper limits of RRs is from 1.63 to 6.11 in Scenarios 3-1 and 3-2 in Table 3. If the expected RR is larger than 1.63, researchers are encouraged to increase the sample size to narrow the baseline incidence 95% CIs and subsequently limit the range of upper limits to avoid obtaining RRs reaching or exceeding the upper limits.

Upper limits to other relative measures

Furthermore, we argue that ORs and rate ratios are also under such constraints. ORs obtained from case-control studies or logit regressions aim to approximate RRs of rare events [1]. Researchers who use ORs for their research should take the upper limits of RRs very seriously. Researchers and research users should think what is the value of providing OR estimates that are likely to be unrealistic and exceed the upper limits of RRs. When the approximation to RRs is outside of the upper limits, we think the ORs reported in such scenarios may merely represent exaggerated effect sizes that are implausible in the real world. Moreover, the 95% CIs of ORs may exacerbate this issue. This is because the 95% CIs of ORs are derived from the exponentiation of a normal approximation [2]. Due to exponentiation, the upper bound of the OR 95% CI can be further away from the OR point estimate than the lower bound in absolute values. This may lead to a higher chance of observing an overlap between OR 95% CIs and the range of RR upper limits derived from baseline incidence 95% CIs.

Moreover, ORs consistently over- or under-estimate the RRs when the underlying RRs are higher or lower than 1, respectively [1,10]. It has been recommended to correct the ORs to obtain a better approximation to RRs when the baseline incidence among individuals not at risk can be estimated [1]. In Figure 1, one of the equations proposed to correct the ORs is listed [1]. This equation can effectively limit the corrected ORs (point estimates) within the upper limits of RRs if the baseline incidence can be estimated [1]. However, the 95% CIs of corrected ORs may still exceed the upper limits of RRs. This is partly because this correction method does not address the uncertainties in the baseline incidence [1].

For ORs reporting in Figure 6, it is possible that the baseline incidence in the population is unknown or cannot be estimated. Without baseline incidence, the ORs cannot be corrected to better approximate RRs using the method in Figure 1 [1] and the upper limits to RRs cannot be estimated. It is recommended to report that the baseline incidence could not be well estimated, and ORs could not be corrected using baseline incidence.

For rate ratios that are calculated by dividing the rates in the at-risk groups by those in the control groups [2], upper limits can be obtained by calculating the multiplicative inverse of the baseline rate in the control group. We also encourage researchers to consider the rate ratio's upper limits and assess whether the 95% CIs could exceed the upper limits when reporting rate ratios. We will assess the overlap between the rate ratio upper limits and 95% CIs in detail.

Limitations

This study does not address the situations in which zero events were observed in the control group [27]. We aim to review the recommendations for studies with zero events in the future [27]. This issue is important because the equation in Figure 1 requires non-zero events in the not-at-risk group. Without disease occurrence in the not-at-risk group, RR could not be calculated. Current recommendations for studies with zero events included reporting absolute risk differences only [28], continuity correction [29], Bayesian methods, and Mantel-Haenszel methods for meta-analysis [30]. We will use the simulation framework in our study to understand the occurrence of zero events and ranges of potential underlying RR. Other measures, particularly risk differences, remain relevant. We are developing methods to quantify the probabilities that the 95% CIs of RRs may exceed the upper limits, given the uncertainties in baseline incidence. More detailed reporting recommendations may be available in the future. In addition, an inherent characteristic of RR is that it is used to describe the strength of the association between a risk factor and a disease. When two risk factors are involved, multiple comparison or intervention groups may be generated for the comparison with the control or baseline group and multiple RRs will be derived to understand the effects of risk factors. In such circumstances, we think the estimates of baseline incidence 95% CIs are likely to expand and influence the estimation of RR upper limits. However, this needs to be tested in further study. Lastly, our recommendations on the reporting of relative measures do not replace other reporting guidelines that focus on study design or the risk of bias or validity of composite measures [31].

## Conclusions

When evaluating the risk of developing a disease due to a risk factor, the upper limit to the RR, the effect size of the risk factor, is the multiplicative inverse of the baseline cumulative incidence of the disease, as shown in equations, simulations, and example scenarios. Moreover, there is a disconnect between RR 95% CIs and upper limits. In the scenarios, we demonstrated RR 95% CIs may exceed the upper limits and researchers can overestimate the effect sizes, if the upper limits are not addressed. The statistical significance of the RR does not guarantee that the 95% CI does not exceed the upper limits. The upper limits have implications on the use and reporting of relative measures, particularly RRs and ORs, and power analysis for sample sizes. We strongly recommend researchers report both absolute and relative effect sizes and calculate the range of upper limits of RR based on the baseline incidence and its 95% CI. This upper limit should be calculated and reported when applicable. Whether the range of upper limits derived from the baseline incidence 95% CIs overlaps with the RR 95% CIs should be determined. The probabilities of RR reaching and exceeding upper limits should be discussed. This upper limit should be considered in power analysis for sample sizes when using expected RRs. The correction of ORs should be considered and reported, especially when the baseline incidence can be estimated. We recommend researchers to report relative measures along with the range of RR upper limits derived from the 95% CIs of baseline incidence. Researchers are recommended to report whether the 95% CIs of relative measures, RRs, ORs, and rate ratios, overlap with the range of upper limits and discuss whether the relative measure estimates may exceed the upper limits.

## Figures and Tables

**Figure 7:**
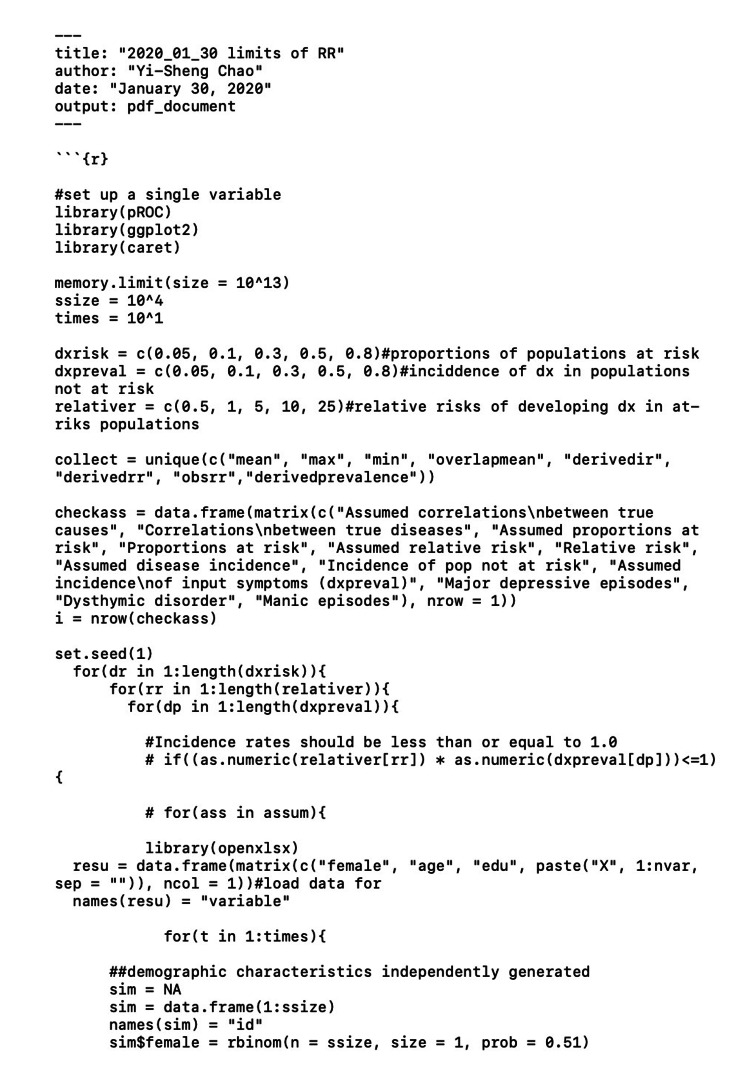
Appendix 1-1

**Figure 8:**
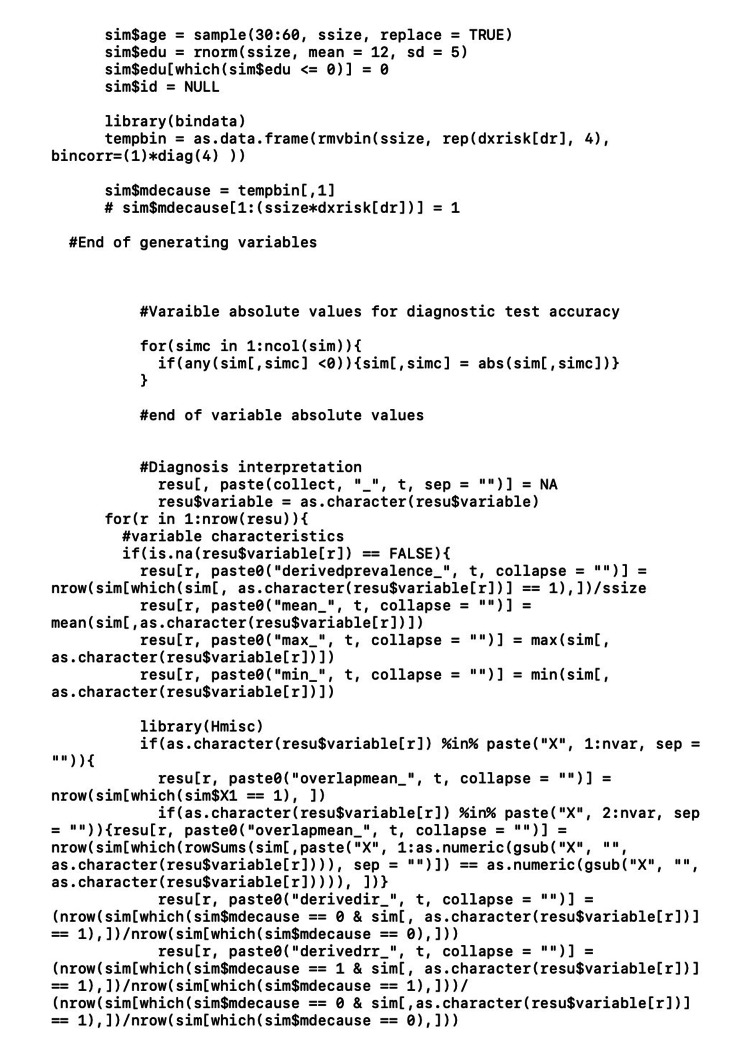
Appendix 1-2

**Figure 9:**
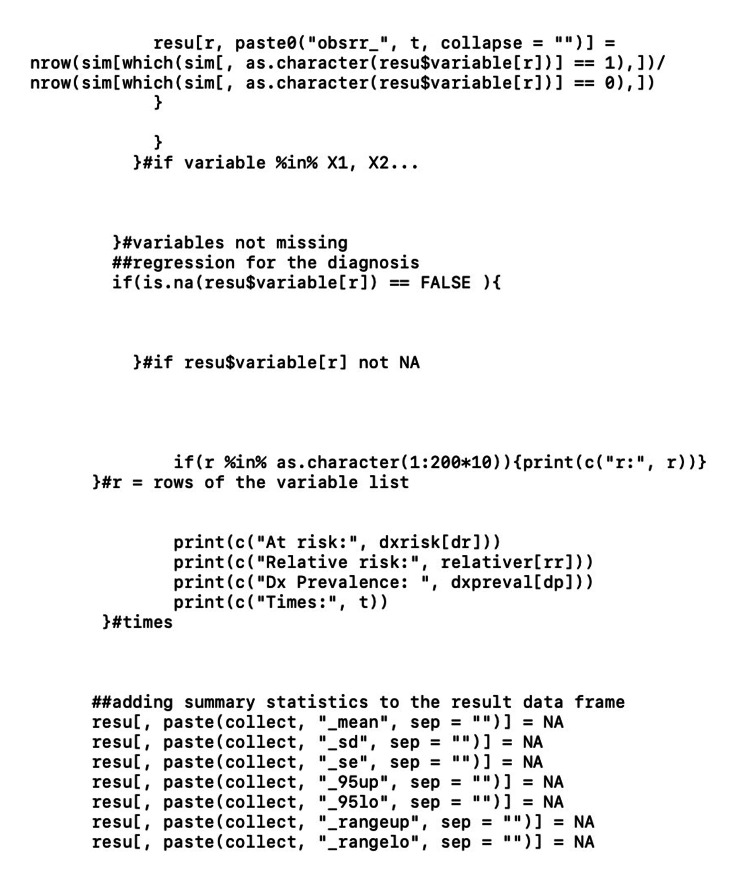
Appendix 1-3

## Appendices

Appendix 1

Appendix 2

**Table 4:** Appendix 2

Proportions at risk	Baseline incidence	Relative risk	Derived overall mean incidence	Derived average baseline incidence	Derived average relative risk	Derived overall disease incidence
0.05	0.05	0.5	0.0468	0.049721054	0.457127618	0.04835
0.1	0.05	0.5	0.0475	0.049897494	0.48805841	0.04735
0.3	0.05	0.5	0.0409	0.050500852	0.492179708	0.04278
0.5	0.05	0.5	0.0356	0.049279621	0.501675888	0.03697
0.8	0.05	0.5	0.0272	0.049034751	0.516032632	0.02966
0.05	0.1	0.5	0.0952	0.09778401	0.500348641	0.0954
0.1	0.1	0.5	0.0947	0.098687161	0.506362136	0.09377
0.3	0.1	0.5	0.0831	0.099307316	0.533478986	0.08538
0.5	0.1	0.5	0.073	0.100566855	0.492586906	0.075
0.8	0.1	0.5	0.0619	0.100416899	0.498282312	0.05987
0.05	0.3	0.5	0.2982	0.301634597	0.523014916	0.29454
0.1	0.3	0.5	0.2883	0.302623249	0.487103393	0.28699
0.3	0.3	0.5	0.2485	0.298621311	0.507464813	0.25415
0.5	0.3	0.5	0.2251	0.302175759	0.494644519	0.2263
0.8	0.3	0.5	0.1862	0.306158093	0.491450992	0.18123
0.05	0.5	0.5	0.4875	0.500100718	0.507790772	0.48793
0.1	0.5	0.5	0.4743	0.49997063	0.504907519	0.47516
0.3	0.5	0.5	0.4315	0.499238159	0.507429164	0.4262
0.5	0.5	0.5	0.3807	0.497404464	0.507006321	0.37405
0.8	0.5	0.5	0.306	0.495011217	0.510778461	0.30133
0.05	0.8	0.5	0.7728	0.799768307	0.491208854	0.77959
0.1	0.8	0.5	0.7568	0.799857756	0.48992427	0.75896
0.3	0.8	0.5	0.6823	0.80034303	0.494939362	0.67947
0.5	0.8	0.5	0.6009	0.798185593	0.505679584	0.60051
0.8	0.8	0.5	0.481	0.796259486	0.502771404	0.47935
0.05	0.05	1	0.0522	0.051483713	0.918815583	0.05128
0.1	0.05	1	0.0519	0.050151279	0.941733838	0.04986
0.3	0.05	1	0.0511	0.050036612	0.997465591	0.04998
0.5	0.05	1	0.0493	0.050055087	0.977082986	0.04944
0.8	0.05	1	0.0471	0.051047916	0.977933279	0.0498
0.05	0.1	1	0.1071	0.099430194	1.005641167	0.09946
0.1	0.1	1	0.1004	0.100640718	1.00402877	0.10068
0.3	0.1	1	0.1005	0.100882749	1.002036598	0.10084
0.5	0.1	1	0.0991	0.099365578	1.004434208	0.09955
0.8	0.1	1	0.1006	0.102555697	0.990285903	0.10136
0.05	0.3	1	0.2986	0.299522554	1.000126214	0.29953
0.1	0.3	1	0.297	0.302366997	0.956920038	0.30107
0.3	0.3	1	0.3021	0.297419153	1.004057194	0.29775
0.5	0.3	1	0.3026	0.299350046	0.991647609	0.29809
0.8	0.3	1	0.2972	0.302925765	0.98865623	0.29992
0.05	0.5	1	0.4981	0.502242638	0.989617518	0.502
0.1	0.5	1	0.5023	0.499440425	1.008085725	0.49985
0.3	0.5	1	0.4938	0.497098448	1.005670184	0.49788
0.5	0.5	1	0.5016	0.499592718	0.998281999	0.49906
0.8	0.5	1	0.5016	0.509821479	0.984109574	0.50323
0.05	0.8	1	0.8009	0.800179193	1.005911643	0.80042
0.1	0.8	1	0.7943	0.799237893	1.006522377	0.79974
0.3	0.8	1	0.8083	0.799437416	1.002305981	0.79995
0.5	0.8	1	0.7971	0.794411888	1.006502765	0.79697
0.8	0.8	1	0.8	0.803615559	0.994505499	0.80006
0.05	0.05	5	0.0592	0.050449313	4.839591682	0.05994
0.1	0.05	5	0.0677	0.04969169	4.945997799	0.06905
0.3	0.05	5	0.1075	0.049039995	5.07386561	0.10901
0.5	0.05	5	0.1469	0.050763928	4.914036481	0.15011
0.8	0.05	5	0.2126	0.050594181	4.925431213	0.20789
0.05	0.1	5	0.1209	0.100534236	4.933250144	0.12036
0.1	0.1	5	0.1402	0.100478178	4.934758294	0.13986
0.3	0.1	5	0.2235	0.10130359	4.998789762	0.22352
0.5	0.1	5	0.3045	0.101015176	4.953601604	0.29968
0.8	0.1	5	0.4218	0.096351854	5.227869639	0.42049
0.05	0.3	5	0.3259	0.29834122	3.352337821	0.33311
0.1	0.3	5	0.3762	0.301038245	3.32231595	0.37132
0.3	0.3	5	0.5026	0.300570063	3.328476186	0.50914
0.5	0.3	5	0.6484	0.302414805	3.307202264	0.6517
0.8	0.3	5	0.861	0.299570633	3.341981615	0.86025
0.05	0.5	5	0.5267	0.502518464	1.990163262	0.52798
0.1	0.5	5	0.5509	0.500722528	1.997271402	0.55077
0.3	0.5	5	0.6592	0.500974587	1.996453094	0.65058
0.5	0.5	5	0.7446	0.500062889	2.000337944	0.74958
0.8	0.5	5	0.8963	0.500135027	2.000393825	0.90008
0.05	0.8	5	0.8125	0.80100817	1.248436878	0.81091
0.1	0.8	5	0.8243	0.799443102	1.250925072	0.81959
0.3	0.8	5	0.8586	0.798550994	1.252331332	0.85895
0.5	0.8	5	0.9027	0.800237643	1.249685456	0.90015
0.8	0.8	5	0.9561	0.797996328	1.253235783	0.95952
0.05	0.05	10	0.078	0.0500067	10.09286843	0.07284
0.1	0.05	10	0.1004	0.052214127	9.367572428	0.09522
0.3	0.05	10	0.1788	0.050522813	9.903648787	0.18484
0.5	0.05	10	0.2732	0.050075347	10.07419943	0.27656
0.8	0.05	10	0.4133	0.049425081	10.20524176	0.40936
0.05	0.1	10	0.1439	0.09901342	10.10735764	0.14334
0.1	0.1	10	0.192	0.099999445	10.01093728	0.191
0.3	0.1	10	0.3675	0.101034627	9.914242614	0.37005
0.5	0.1	10	0.5575	0.099389883	10.07159299	0.55013
0.8	0.1	10	0.8205	0.097855113	10.2807742	0.81694
0.05	0.3	10	0.3337	0.301407263	3.318963297	0.33689
0.1	0.3	10	0.3756	0.301937882	3.312592096	0.37103
0.3	0.3	10	0.5048	0.29964453	3.337630208	0.51013
0.5	0.3	10	0.6579	0.300211139	3.332929265	0.64898
0.8	0.3	10	0.8626	0.30145048	3.318442296	0.86051
0.05	0.5	10	0.5303	0.501630703	1.99367313	0.52634
0.1	0.5	10	0.5527	0.500414953	1.99856783	0.55051
0.3	0.5	10	0.6559	0.501507291	1.994364645	0.65221
0.5	0.5	10	0.7541	0.499178956	2.003552801	0.74953
0.8	0.5	10	0.9015	0.496092771	2.016573003	0.89919
0.05	0.8	10	0.8154	0.801568356	1.247571637	0.81141
0.1	0.8	10	0.8181	0.800036559	1.249947568	0.81983
0.3	0.8	10	0.8618	0.800242671	1.249642646	0.86032
0.5	0.8	10	0.9004	0.795093594	1.257785208	0.89808
0.8	0.8	10	0.9554	0.799088597	1.251564965	0.95991
0.05	0.05	25	0.0974	0.050379705	19.8693847	0.09711
0.1	0.05	25	0.1459	0.049320074	20.30602453	0.14471
0.3	0.05	25	0.3317	0.050312546	19.97908388	0.33435
0.5	0.05	25	0.5234	0.048283642	20.78352472	0.52336
0.8	0.05	25	0.8117	0.050154497	20.11561068	0.81213
0.05	0.1	25	0.1423	0.099615069	10.04582564	0.14533
0.1	0.1	25	0.193	0.098437058	10.16817989	0.1883
0.3	0.1	25	0.3629	0.100396125	9.977788758	0.36996
0.5	0.1	25	0.5553	0.098871605	10.12704266	0.54767
0.8	0.1	25	0.8181	0.098209825	10.24767005	0.81725
0.05	0.3	25	0.3388	0.300078496	3.332996099	0.33554
0.1	0.3	25	0.3666	0.297902943	3.358020372	0.36816
0.3	0.3	25	0.5067	0.299261926	3.34229051	0.50893
0.5	0.3	25	0.6536	0.299458723	3.34023926	0.64996
0.8	0.3	25	0.8557	0.298799341	3.349401054	0.8596
0.05	0.5	25	0.5219	0.498945186	2.004376922	0.52393
0.1	0.5	25	0.5463	0.497502784	2.010208867	0.54768
0.3	0.5	25	0.6514	0.499864565	2.000706467	0.64879
0.5	0.5	25	0.7464	0.499803655	2.001097784	0.7494
0.8	0.5	25	0.9013	0.49568834	2.017960488	0.89931
0.05	0.8	25	0.8082	0.800682631	1.248993602	0.8105
0.1	0.8	25	0.8219	0.799167884	1.251331098	0.81929
0.3	0.8	25	0.8618	0.799165693	1.251336713	0.85922
0.5	0.8	25	0.8997	0.799416085	1.251029445	0.90003
0.8	0.8	25	0.9614	0.800772152	1.248924631	0.96008
